# Spatial Frequency Multiplexing of Fiber-Optic Interferometric Refractive Index Sensors Based on Graded-Index Multimode Fibers

**DOI:** 10.3390/s120912377

**Published:** 2012-09-12

**Authors:** Li Liu, Yuan Gong, Yu Wu, Tian Zhao, Hui-Juan Wu, Yun-Jiang Rao

**Affiliations:** 1 Key Lab of Optical Fiber Sensing & Communications (Education Ministry of China), University of Electronic Science & Technology of China, Chengdu, Sichuan 611731, China; E-Mails: aya0626@163.com (L.L.); wuyuzju08@gmail.com (Y.W.); junya1987@163.com (T.Z.); hjwu@uestc.edu.cn (H.-J.W.); yjrao@uestc.edu.cn (Y.-J.R.); 2 State Key Laboratory of Transient Optics and Photonics, Xi'an Institute of Optics and Precision Mechanics, Chinese Academy of Sciences, Xi'an, Shaanxi 710119, China

**Keywords:** fiber-optic sensors, graded-index multimode fiber (GI-MMF), Fabry-Perot sensor, refractive index sensor, spatial frequency multiplexing

## Abstract

Fiber-optic interferometric sensors based on graded-index multimode fibers have very high refractive-index sensitivity, as we previously demonstrated. In this paper, spatial-frequency multiplexing of this type of fiber-optic refractive index sensors is investigated. It is estimated that multiplexing of more than 10 such sensors is possible. In the multiplexing scheme, one of the sensors is used to investigate the refractive index and temperature responses. The fast Fourier transform (FFT) of the combined reflective spectra is analyzed. The intensity of the FFT spectra is linearly related with the refractive index and is not sensitive to the temperature.

## 1. Introduction

The refractive index (RI) is a key parameter to evaluate the characteristics of a variety of biochemical species. Via precise refractive index measurement, other properties such as liquid concentration, the gas density and ambient temperature can be determined. Therefore, the precise measurement of refractive index is very important. Fiber-optic Fabry-Perot sensors have been developed as extremely high-precision sensing devices for precise measurement of RI [1,2], temperature [3–6], strain [6,7], pressure [8], and displacement [9], *etc*. Fiber-optic sensors have the advantages of high sensitivity, small size, resistance to electromagnetic interference, fast response, potential for multiplexing and high tolerance to harsh environments, *etc*. Various RI sensors have been developed based on different fiber-optic schemes, such as photonic crystal fiber (PCF) [10], fiber tapers [11–13], multimode interferometric sensors [13,14], fiber Bragg grating (FBG) sensors [15–18], long period fiber gratings (LPFGs) [19,20], and fiber-optic Fabry-Perot sensors [1,2,21]. Among them, fiber-optic interferometric RI sensors have been widely used owing to their properties of high precision and ease of fabrication.

Multiplexing can achieve multi-point simultaneous detection, large area detection, and it can further reduce system costs. The extrinsic fiber Fabry-Perot interferometric sensors (EFPIs) have many advantages, but it is difficult to multiplex multiple sensors and this leads to a high average cost of single sensors [22]. The spatial frequency division multiplexing method [23] can increase the number of sensors used in one fiber-optic sensing system, thus reducing the cost. Lee and co-workers have reported a temperature and refractive index Fabry-Perot interferometric sensor [1], and used it for hydrogen gas measurement [24]. In this paper, the multiplexing capability of fiber-optic interferometric sensors based on the graded-index multimode fibers (GI-MMFs) is estimated. The refractive index and temperature responses of such sensors are tested under multiplexing condition. In addition, the principle of such sensors is further investigated by analyzing the spot distribution on the fiber end via a near-field optical profiler.

## 2. Principle of Fiber-Optic Interferometric Sensor Based on GI-MMF

**Figure 1.**
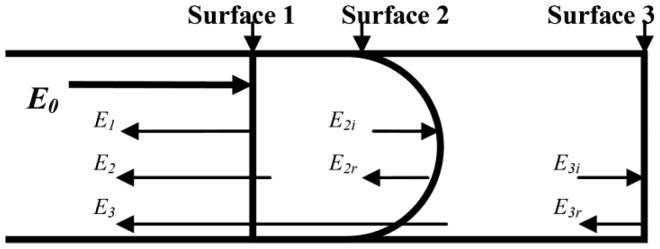
Schematic of the fiber-optic three-beam interferometric sensor.

The sensors investigated by this paper are based on the periodic focusing effect of GI-MMFs and the three-beam interferometer [25,26]. The GI-MMF has a core diameter of 62.5 μm with high Ge doping concentration, and the numerical aperture was 0.275. Firstly, the GI-MMF was chemically etched by 40% hydrofluoric acid solution. This process is quite simple and cost-effective. The etching time was about one and a half minutes to obtain a microscale hole on the fiber tip. The etched fiber tip was fusion spliced to a single-mode fiber to form an air cavity. Then the GI-MMF was carefully cleaved to form the third reflective surface, as shown in Figure 1.

The principle of the sensor can be described by the ray transfer matrix (RTM) method [25,27]. The normalized reflected light intensity of the three-beam interference can be expressed as [25]:

(1) $$I_{r}/I_{0}={\left|E_{1}+E_{2}+E_{3}\right|}^{2}/\left|E_{0}{|}^{2}=R_{1}+R_{2\text{eff}}+R_{3\text{eff}}-2\sqrt{R_{1}R_{2\text{eff}}}\cos\phi_{1}+2\sqrt{R_{2\text{eff}}R_{3\text{eff}}}\cos\phi_{2}-2\sqrt{R_{1}R_{3\text{eff}}}\cos(\phi_{1}+\phi_{2}\right)$$

Where *E_0_* is the electric field component of incident light; *E_1_*, *E_2_* and *E_3_* are electric field components of the reflected light of the three surfaces that re-coupled into the single-mode fiber, respectively. *R_1_*, *R_2eff_*, and *R_3eff_* denote the effective reflectivity of the three reflecting surfaces, respectively. *R_3eff_* is directly related to the refractive index of the outside environment. Effective reflectivity relates to reflectance of every surface, transmittance, cavity loss, and coupling efficiency. *φ*_1_ and *φ*_2_ represent respectively the corresponding phase difference related to the air gap and the GI-MMF. The GI-MMF has a large numerical aperture, and its maximum core refractive index is n_1_ = 1.475. Based on the Fresnel equation, the reflectivity of the bottom of the air cavity is about 3.70%. Enhancement of the reflectivity can improve the contrast of the reflective spectra. Because of the periodic focusing effect of the GI-MMF, it can greatly improve the contrast of the interference fringes, thereby increasing the measurement accuracy.

**Figure 2.**
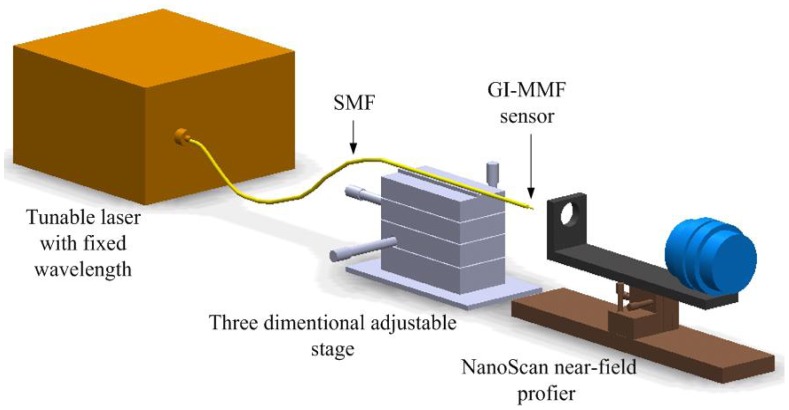
Schematic diagram of experimental setup for the spot size measurement.

**Figure 3.**
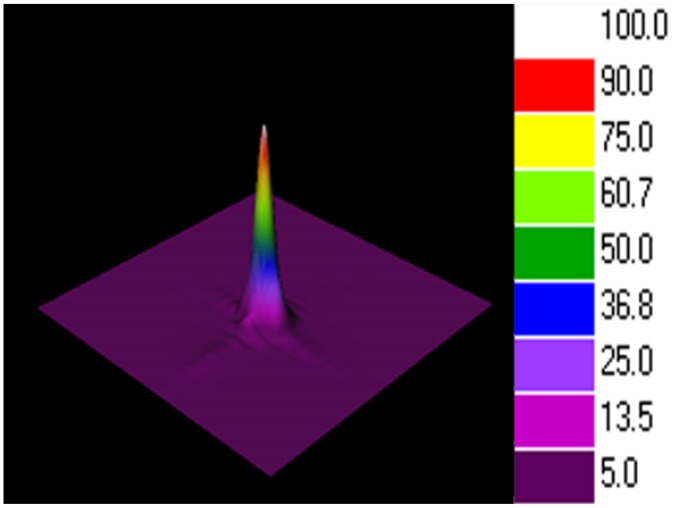
The light intensity distribution on the fiber end of the sensor by a near-field optical profiler.

In order to further investigate the principle of the fiber-optic three-beam interferometric sensors based on the periodic focusing effect of GI-MMF, we fabricated more than 200 such sensors and measured the intensity profile on the fiber end of the third surface, to confirm the periodic spot size distribution on the fiber end. Figure 2 shows the schematic diagram of the mode field analysis system. A wavelength-swept laser was tuned to a wavelength of 1,550 nm, a typical wavelength in the optical communication field, and transmitted through a single mode fiber to the sensing head. The spot size on the fiber end of the sensor was measured with a near-field optical profiler (Model NS-GE/9/5, NanoScan, Ophir Optronics Inc., Jerusalem, Israel). The sensor head is fixed on a three-dimensional adjustable stage so that it can align with the lens. The spot scanning instrument is a NanoScan near-filed profiler, which provides a software to observe the spot sizes by both two-dimensional (2D) and three-dimensional (3D) views. Figure 3 shows a 3D view of the light intensity distribution on the fiber end of the sensing head. Both the color and height refer to the light intensity. The spot size was determined by the full width at half magnitude (FWHM) of the light distribution at the fiber end.

**Figure 4.**
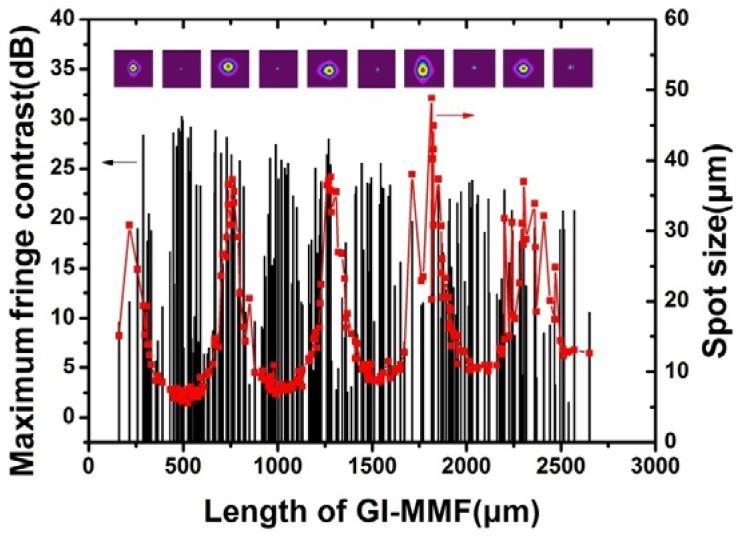
Spot sizes of 212 sensors with different GI-MMF lengths (the red line) and relationship between maximum fringe contrasts and GI-MMF lengths (the black bar).

Figure 4 shows the spot sizes and maximum fringe contrast of 212 sensors with different GI-MMF lengths. The spot size changes periodically as the GI-MMF lengths increases, represented by red dots. The period is about 520 μm. Typical light spots on the fiber end with periodic minimum or maximum radii are also shown in the inset of Figure 4. Figure 4 also shows the relationship between the maximum fringe contrasts and the GI- MMF lengths, denoted by the narrow black bars. It indicates that the maximum fringe contrasts change periodically as the GI-MMF lengths increases. The period is approximate half of the period of the spot sizes. That is, high fringe contrast can be obtained when the GI-MMF length equals the integers of one quarter pitch.

## 3. Multiplexing of Fiber-Optic Interferometric Sensor Based on GI-MMF

**Figure 5.**
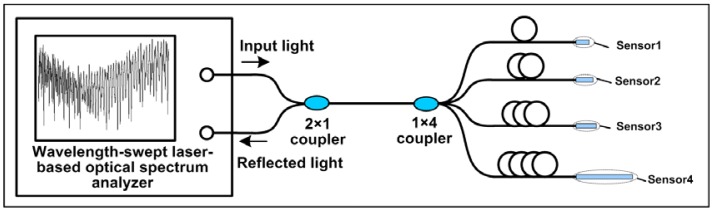
Schematic diagram of the multiplexing system.

**Figure 6.**
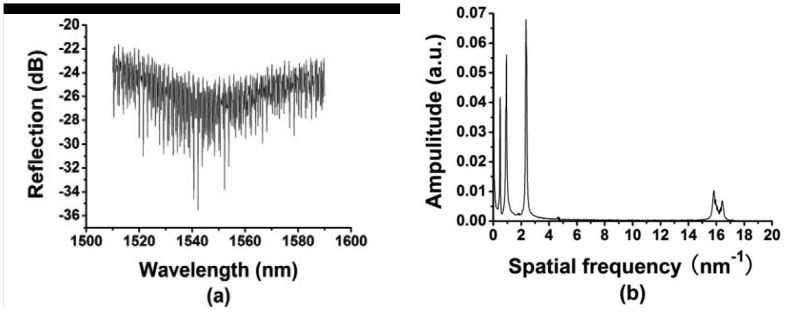
(**a**) Reflected spectrum of four multiplexed sensors with different length of GI-MMF and (**b**) corresponding FFT spectrum.

The schematic diagram of the multiplexing system for the fiber-optic interferometric sensors is shown in Figure 5. Light from an optical spectrum analyzer (OSA) based on the wavelength-swept laser was launched into a 1 × 2 coupler with 50:50 splitting ratios, and then reaches a 1 × 4 coupler with equal splitting ratios. Light was reflected by four sensors, respectively and collected by the OSA. As the optical spectrum analyzer was based on a narrowband swept laser, the single-mode fibers with different length were added to each branch of the 1 × 4 coupler to avoid interference among the reflected light of four sensors. It can also be obtained by using broadband light source with very short coherent length. The reflected spectrum of the four multiplexed sensors in the air was shown in Figure 6(a). Compared with those described in [1] and [24], the air cavity used in our experiment is very short, so the FSR of the air cavity is large. We chose the flat part of the whole spectra, from 1,550 nm to 1,590 nm, to do the data processing, which is equivalent to filter out the FFT peak corresponding to the air cavity, as shown in Figure 6(b). The amplitude of the FFT spectrum is normalized by the peak intensity with a spatial frequency of zero. There are totally four peaks, and each peak represents a sensor used in the experiment. In experiment the refractive index sensors have GI-MMF lengths of 385, 760, 1,970 and 14,000 μm, respectively, measured by an optical microscope. When the GI-MMF length gradually increases, more than one mode is excited, and there is a slight difference among the corresponding optical paths, resulting that periodicity of the reflectance spectra was partially damaged, and ultimately forming the peak broadening and frequency splitting of FFT spectra, see the fourth peak in Figure 6(b).

**Figure 7.**
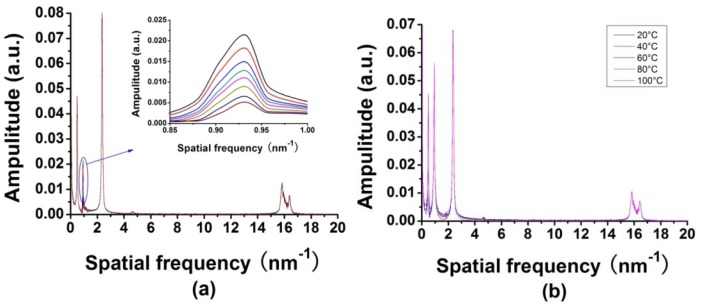
(**a**) FFT of the reflective spectrum of the four sensors with one dipped in liquid with different refractive indices and (**b**) with the same sensor put in different temperature.

**Figure 8.**
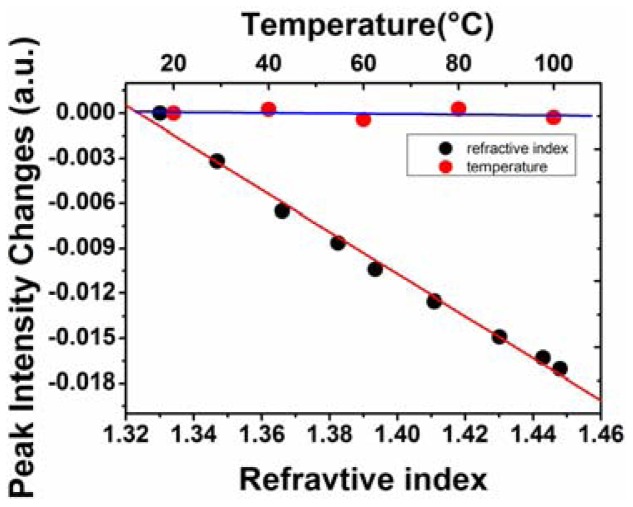
Peak intensity changes as a function of the external refractive index (black) and ambient temperature (red).

The refractive index response of the fiber-optic interferometric sensor was investigated under the multiplexing conditions. The solution used in the refractive index experiment was syrup solution. The refractive index of the solution was changed by adding different amounts of the saturated syrup solution to deionized water. The RIs of solutions with different concentrations were calibrated by an Abbe refractometer. The refractive index of deionized water was about 1.33, and the maximum refractive index in the experiment was about 1.448. The measurement was performed at room temperature. Dipping the sensor in the solutions with different refractive indices, the FFT of the reflective spectrum of the tested sensor would change significantly, as shown in Figure 7(a). The tested sensor had a GI-MMF length of 760 μm, corresponding to the second peak of the FFT spectrum. As the refractive index increases, the intensity of the second peak became smaller, as shown in the inset of Figure 7(a). Similarly, the temperature responses were also measured. The FFT spectrum was shown in Figure 7(b). In the experiment, the temperature varied from 20 °C to 100 °C at intervals of 20 °C. The peak intensities of the FFT spectrum were not sensitive to the temperature changes. The intensity changes of the second peak in the FFT spectrum as a function of the external refractive index and ambient temperature are shown in Figure 8. The linear fits are also given. A linearity of −0.99634 and a refractive index coefficient of about −0.14036 per refractive index unit (RIU) was obtained. In temperature experiment, it shows that the peak intensity of the FFT of the second sensor changes 0.00068 with the temperature changes from 20 °C to 100 °C. The temperature sensitivity is about 8.5 × 10^−6^/°C. Thus the temperature cross sensitivity is 6.0 × 10^−5^ RIU/°C, which is negligible and the sensor can be considered temperature-insensitive.

As the length of the GI-MMF increases, the total loss increases and the higher order modes would be excited in the GI-MMF. The fringe contrast of the sensor's reflective spectrum also decreases with increasing the GI-MMF length and thus the intensity of the corresponding FFT peak becomes weak, for example, the fourth sensor with 14,000 μm-length GI-MMF in our experiment. Finally the accuracy of the refractive index measurement using the sensor with longer length GI-MMF decreases.

## 4. Conclusions

The spatial multiplexing of the fiber-optic three-beam interferometric refractive index sensors has been investigated. Fiber-optic three-beam interferometric sensors with different GI-MMF lengths have been fabricated. The periodic focusing effect of the GI-MMF has been investigated in detail with a near-field optical profiler. In order to prove the multiplexing performance of this kind of sensor, one sensor with a long GI-MMF length of 14,000 μm has been fabricated. It is indicated by the experimental results that this kind of sensor has good multiplexing capability and is not sensitive to temperature.
